# Improved Anti-Oxidant and Anti-Bacterial Capacities of Skim Milk Fermented by *Lactobacillus plantarum*

**DOI:** 10.3390/molecules29163800

**Published:** 2024-08-10

**Authors:** Ying Wang, Bingtian Zhao, Yun Ding, Nan Liu, Cheng Yang, Yajuan Sun

**Affiliations:** 1 Key Laboratory of Synthetic and Biological Colloids, Ministry of Education, School of Chemical and Material Engineering, Jiangnan University, Wuxi 214122, China; wy2133590196@163.com (Y.W.); btzhao@jiangnan.edu.cn (B.Z.); cyang@jiangnan.edu.cn (C.Y.); 2 Hangzhou Island Xingqing Biotechnology Co., Ltd., Hangzhou 310023, China

**Keywords:** skim milk, protease hydrolysis, fermentation, anti-oxidant, anti-bacterial

## Abstract

Milk, on account of its abundant protein content, is recognized as a vital source of bioactive substances. In this study, the bioactive ingredients in milk were obtained by a combination of protease hydrolysis and fermentation with *Lactobacillus plantarum*. The compositions of protease hydrolysate (PM) and fermentation supernatant (FM) were determined, and their anti-oxidant and anti-bacterial activities were evaluated. Using LC-MS/MS, the molecular weights and sequences of the peptides were characterized, among which a total of 25 bioactive peptides were identified. The DPPH radical scavenging results demonstrated that FM exhibited an enhanced anti-oxidant capacity compared to PM. The bacterial survival rate results revealed that FM had a remarkable anti-bacterial ability compared to PM. Additionally, the anti-bacterial component and potential anti-bacterial mechanisms were determined. The results of cytoplasmic membrane depolarization, cell membrane permeability, and morphological observation indicated that FM could interact with bacterial membranes to achieve its anti-bacterial effect. These findings suggested that FM, as a bioactive substance of natural origin, holds potential applications in the functional food, pharmaceutical, and cosmetic industries.

## 1. Introduction

Milk is widely recognized as a rich source of natural bioactive ingredients, which provides multiple nutrients for humankind, including casein, whey protein, and so on [1]. Casein constitutes approximately 80% of milk protein and plays a significant role in anti-thrombus, anti-oxidant, and immunoregulation activities [2]. Lactoferrin, a key component of whey protein, is considered a vital host defense molecule due to its ability to inhibit the growth of pathogenic bacteria [3]. However, these active components are encoded within the parent protein, which limits their applications in the pharmaceutical and cosmetic industries [4,5].

Fermentation, a common approach used to release the active components of proteins, refers to taking advantage of bacteria that can produce proteases to hydrolyze these proteins [6]. During fermentation, probiotics not only release active ingredients from the substrate and enhance their levels through metabolism, but also reduce the toxicity [7,8]. Begunova et al. revealed that cow’s milk fermented with *Lactobacillus helveticus* NK1, *Lactobacillus rhamnosus* F, and *Lactobacillus reuteri* LR1 exhibited anti-oxidant and anti-hypertensive properties [9]. Moreover, microorganisms are able to convert glucose and glycerol in the substrate into organic acids, fatty acids, and volatile compounds, which work synergistically with proteins to enhance moisturizing, anti-bacterial, and anti-fungal activities [10,11]. The supernatant from the fermentation of skim milk by *Streptococcus thermophilus* YIT 2084 demonstrated an outstanding skin hydration effect, protected skin cells from oxidative stress, and maintained the acid–base balance of the skin due to the presence of hyaluronic acid, lactic acid, and amino acids [12].

However, the probiotics exhibit great differences in proteolytic activity and peptide utilization [13]. A previous study reported that the proteolytic capability of *Lactobacillus plantarum* is limited, which led to a long fermentation time and high production cost, and the mechanism is indistinct [14]. Therefore, the purpose of this study was to extract the bioactive ingredients in skim milk through a combination of proteolytic hydrolysis and fermentation with *Lactobacillus plantarum*. The synergistic effect of the two methods was expected to improve the efficiency of probiotics, shorten the fermentation time, and diversify biological activities [15]. Based on this, this study investigated the change in the composition of peptides, the anti-oxidant and anti-bacterial bioactivities, and the mechanism of anti-bacterial activity. It is expected that this research can provide a worthwhile scheme for the preparation of active ingredients from natural sources and application in the food, pharmaceutical, and cosmetic industries.

## 2. Results

### 2.1. Identification and Quantification of Potential Active Ingredients

#### 2.1.1. Composition Analysis of Protease Hydrolysate (PM) and Fermentation Supernatant (FM)

The fermentation substrates used in this study included protease-hydrolyzed milk, glucose (2%), yeast powder (0.5%), and 5% *Lactobacillus plantarum* seeds, and the fermentation process was conducted at 37 °C for 12 h with the pH maintained at 5.5 throughout. FM was obtained by centrifuging the fermentation broth and subsequently filtering it through a 0.22 μm membrane.

As shown in Table 1, the peptide was the primary component in PM and FM, with a similar content, accounting for 39.94 ± 1.74% in PM and 30.76 ± 1.95% in FM, respectively. Following fermentation, organic acids were produced, with a proportion of 33.73 ± 2.83% in FM, while their content in PM was undetectable.

#### 2.1.2. Molecular Weight Distribution and Peptidomics of Peptides

**Figure 1:**
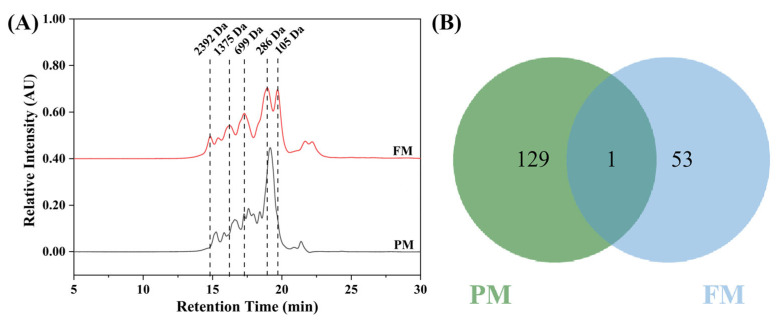
(**A**) The molecular weight distributions and (**B**) Venn diagram of PM and FM.

The molecular weight distribution and peptidomics of peptides were used to assess the degree of peptide hydrolysis and to identify the sequence differences between the peptides in PM and FM. As illustrated in Figure 1A, the presence of proteases led to the extensive hydrolysis of milk proteins, resulting in the production of peptides with a molecular weight of less than 3000 Da [16]. Interestingly, after fermentation, the content of peptides with a molecular weight of less than 300 Da was significantly increased.

Sequence analysis of the peptides in PM and FM was performed using peptidomics. A Venn diagram illustrated that the numbers of peptides in PM and FM were 130 and 54, respectively, and only one identical peptide between them was found (Figure 1B). Moreover, according to the PeptideRanker database, peptides in FM with a prediction score over 0.5 were regarded as bioactive, among which a total of 25 peptides met this criterion (Table S1). The BIOPEP database illustrated that 16 of these bioactive peptides possessed anti-oxidant and anti-bacterial properties (Table 2), accounting for 60.96% of the total peptides in FM and originating from three types of proteins, including β-casein, α-S2-casein, and α-lactalbumin.

### 2.2. Anti-Oxidant Activity

**Figure 2:**
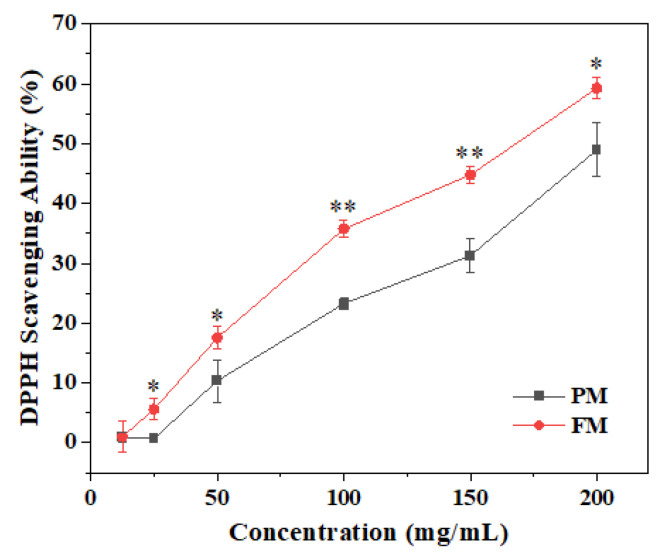
The DPPH scavenging ability of PM and FM. * *p* < 0.05 and ** *p* < 0.01 indicate that the DPPH scavenging capacity of FM was significantly and extremely significantly higher than that of PM, respectively.

A DPPH assay was performed to determine the anti-oxidant ability. Figure 2 shows that the anti-oxidant behavior of PM and FM both showed concentration-dependent characteristics. The DPPH scavenging rate of FM reached from 1.04 ± 2.51% at 12.5 mg/mL to 59.25 ± 1.84% at 200 mg/mL, which was higher than that of PM under the same concentration.

### 2.3. Anti-Bacterial Activity

#### 2.3.1. Minimum Inhibitory Concentration (MIC)

In this study, *Staphylococcus aureus* representing Gram-positive bacteria and *Escherichia coli* representing Gram-negative bacteria were selected to identify the anti-bacterial activity. MIC values of FM for *Staphylococcus aureus* and *Escherichia coli* were 200 mg/mL and 150 mg/mL, respectively (Table 3).

#### 2.3.2. Bacterial Survival Analysis

**Figure 3:**
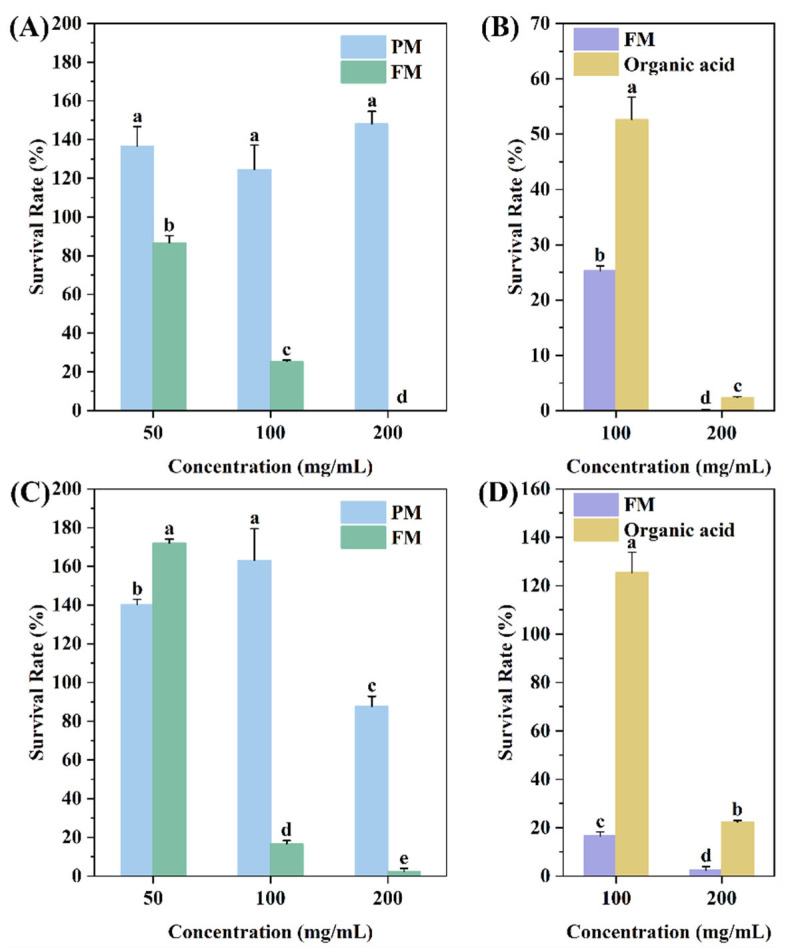
The bacterial survival rate of *Staphylococcus aureus* (**A**,**B**) and *Escherichia coli* (**C**,**D**) after 24 h of incubation with PM or FM at the final concentrations of 50, 100, and 200 mg/mL (**A**,**C**), with 100 and 200 mg/mL of FM or corresponding concentrations of organic acid (**B**,**D**). Different letters (a–e) indicate significant differences.

The bacterial survival rates of *Staphylococcus aureus* and *Escherichia coli* cultivated with 50, 100, and 200 mg/mL of PM/FM for 24 h are shown in Figure 3A,C. PM with different concentrations promoted the growth of both strains, while FM reduced the viability of the two pathogens in a concentration-dependent manner. When the concentration of FM reached 200 mg/mL, the survival rates of *Staphylococcus aureus* and *Escherichia coli* were 0 and 2.39 ± 1.50%, respectively. These results demonstrated that fermentation significantly enhanced the anti-bacterial capacity of FM.

To identify the anti-bacterial constituents in FM, the bacterial survival rate of FM and organic acids at the corresponding concentrations was examined (Figure 3B,D). The results revealed that the anti-bacterial activity of pure organic acids was inferior to that of FM at the same concentration. Considering the composition of FM, the peptidomics analysis, and the prediction results, it can be concluded that the anti-bacterial effect of FM is mainly attributed to the peptides. Hence, the anti-bacterial activity of FM is likely due to the synergistic action of peptides and organic acids.

#### 2.3.3. Cytoplasmic Membrane Depolarization

**Figure 4:**
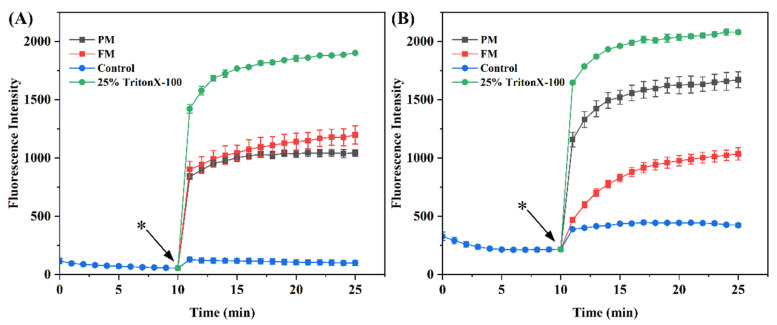
The cytoplasmic membrane potential of *Staphylococcus aureus* (**A**) and *Escherichia coli* (**B**) treated with samples. (*) indicates sample addition.

The dye DiSC3 (5), which adsorbs onto bacteria with an intact cytomembrane, resulting in fluorescence quenching and fluorescing when the membrane is damaged, was used to assess whether FM could induce membrane depolarization. As shown in Figure 4, the fluorescence intensity of DiSC3 (5) decreased gradually as the dye accumulated in the intact membrane. After adding the samples at the 10th minute, the fluorescence intensity of PM and FM both showed a rapid increase compared with the control group, and progressively enhanced as time went on. This could be due to the collapse of the ion gradient, which led to changes in the membrane potential. For this experiment, 25% TritonX-100, which could completely depolarize the membrane, was used as the positive control [17]. As expected, the fluorescence intensity of 25% TritonX-100 was greater than those of PM and FM. It should be noted that the fluorescence intensity of PM and FM for *Staphylococcus aureus* and *Escherichia coli* differed. For *Staphylococcus aureus*, the intensity of FM was slightly higher than that of PM; conversely, FM presented a much lower intensity than PM for *Escherichia coli*. This variation could be ascribed to the samples, which could affect the fluorescence intensity of DiSC3 (5). To verify this hypothesis, the fluorescence intensity of samples with dye in the absence of bacteria was detected and is shown in Table 4. It could be found that the fluorescence intensity of PM with dye was much higher than that of the FM and control groups, which agreed with the hypothesis. The increased intensity was probably due to the dye, which is sensitive to the local changes in solvent, resulting in enhanced fluorescence [18]. After removing the influence of samples, FM showed a higher fluorescence intensity than PM for both bacteria, which agreed with the anti-bacterial activity of FM and PM. Hence, the anti-bacterial activity of FM may be related to its ability to induce membrane depolarization.

#### 2.3.4. Bacterial Cell Membrane Permeability

**Figure 5:**
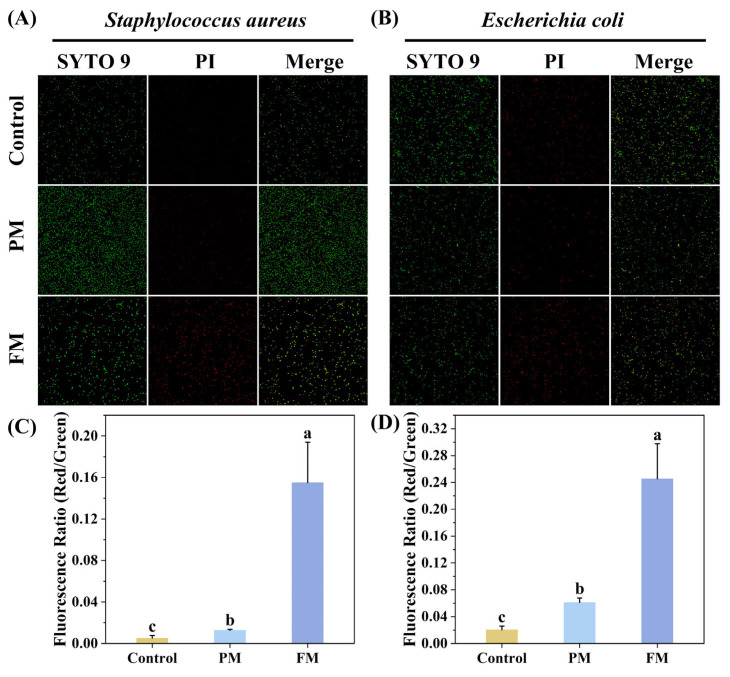
Live–dead fluorescence microscope images from *Staphylococcus aureus* (**A**) and *Escherichia coli* (**B**) with 200 mg/mL of PM and FM. The ratio of red fluorescence intensity (dead bacteria) to green fluorescence intensity (live bacteria) of *Staphylococcus aureus* (**C**) and *Escherichia coli* (**D**). The different superscript letters (a–c) indicate that the results possessed significant differences (*p* < 0.05).

The effects of the PM and FM on bacterial cell membrane permeability were confirmed using the dyes SYTO 9 and PI. SYTO 9 could dye all cells, while PI specifically identifies the damaged cell membranes. From the staining results of PI in Figure 5A,B, it could be observed that a great deal of *Staphylococcus aureus* and *Escherichia coli* died following the treatment with FM, while no such phenomenon was observed in the control and PM groups. The result of the PI/SYTO 9 fluorescence intensity ratio revealed that the PM group exhibited a slightly higher ratio than the control group, suggesting that PM had a minimal effect on cell membrane permeability. In contrast, the PI/SYTO ratio in the FM group increased approximately elevenfold compared to the control group, indicating that the integrity of the cell membrane was damaged severely (Figure 5C,D). These results demonstrated that FM could kill *Staphylococcus aureus* and *Escherichia coli* by enhancing the permeability of their membranes.

#### 2.3.5. Morphological Observation of Bacteria

**Figure 6:**
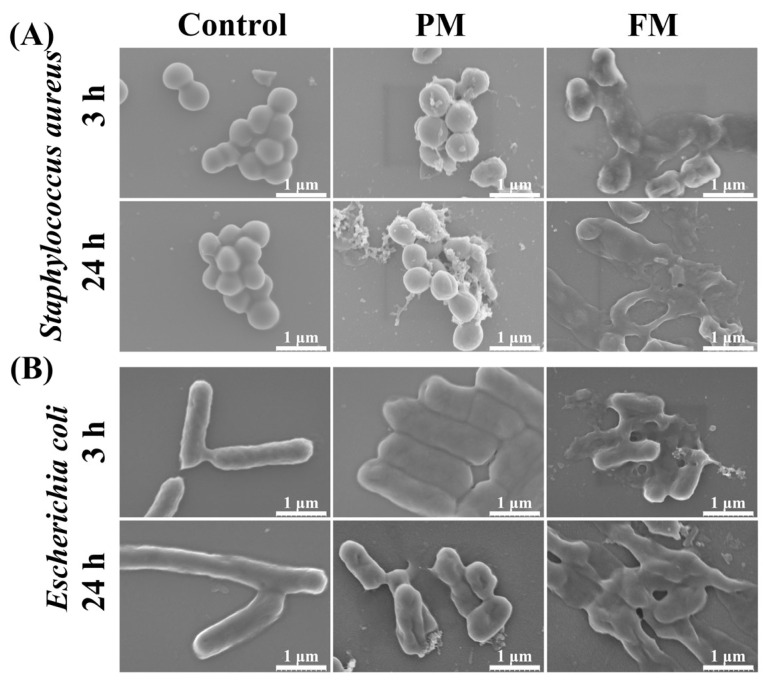
SEM images of *Staphylococcus aureus* (**A**) and *Escherichia coli* (**B**) in the absence and presence of PM or FM.

The changes in surface morphology of *Staphylococcus aureus* and *Escherichia coli* treated with PM/FM were observed by scanning electron microscopy (SEM) (Figure 6). In the absence of PM/FM, the surface of the bacteria was smooth and wrinkle-free. However, after treatment with FM for 3 h, pores and collapse appeared on the surface of some bacteria. When the treatment time was lengthened to 24 h, the bacterial surface was severely crumpled and collapsed, and numerous bacteria shriveled as well as lost their original morphology, indicating abnormal cell rupture and cytoplasmic leakage. The damage degree of bacterial morphology was aggravated over the treatment time with FM. When treated with PM for the same time, some bacterial morphology changed as well, but the damage degree was significantly less than that of the FM group.

## 3. Discussion

This study explored the active ingredients in fermented milk and evaluated the anti-oxidant and anti-bacterial activities. In the course of the *Lactobacillus plantarum* growth, glucose was consumed and converted into organic acids, and high-molecular-weight polypeptides transformed into peptides with a much smaller molecular weight and oligopeptides. Cui et al. indicated that the bioactivity of peptides was negatively correlated with their molecular weight [19]. Hence, it was significant to study the biological activities of FM.

The DPPH radical scavenging rate of PM and FM revealed that the anti-oxidant activity of FM was superior to that of PM. The anti-oxidant levels of milk are closely associated with the bioactive peptides [20]. Proteolytic hydrolysis could produce more anti-oxidant peptides; hence, fermentation with probiotic strains could further enhance anti-oxidant capacity.

In the present investigation, it could be found that FM exhibited anti-bacterial activity against the tested pathogens, *Staphylococcus aureus* and *Escherichia coli*, as evidenced by the MIC and bacterial survival rate results. In contrast, PM was found to promote the growth of these bacteria. The anti-bacterial activity of fermented milk was also reported by other researchers. Cirrincione et al. indicated that ass milk fermented with *Lactococcus lactis subsp. cremoris* 40FEL3 and *Lactobacillus rhamnosus* 17D10 produced bioactive peptides, which reduced *Escherichia coli* growth and inhibited the replication of HSV-1 [21]. Pihurov et al. revealed that milk kefir grains fermented with Kombucha culture possessed anti-fungal and anti-bacterial activity because of the generation of organic acids [22]. Overall, anti-bacterial compounds generated during fermentation mainly include anti-bacterial peptides and organic acids [23]. Combined with the composition of FM, peptidomics analysis, and the anti-bacterial results, the anti-bacterial capacity of FM was likely due to the synergistic effect of peptides and organic acids produced during the *Lactobacillus plantarum* fermentation.

Bacteria comprise the cytomembrane, cytoderm, cytoplasm, and karyoplasm, among which the cytomembrane plays a vital role in facilitating energy and matter exchange between bacteria and the external environment, as well as maintaining normal physiological and metabolic functions [24]. When the cytomembrane is subjected to damage, typical features, involving alterations in membrane potential, compromised membrane integrity and permeability, the leakage of intracellular proteins and nucleic acids, and abnormal morphological alterations, will emerge [24,25]. To explore the effect of FM on the cytomembrane, the changes in membrane potential were first observed using DiSC3 (5). Similar to Triton X-100, FM treatment resulted in depolarization of the cytomembrane (Figure 4). Subsequently, the effects of FM on the permeability of the cytomembrane were verified by a live/dead assay. A great deal of dead *Staphylococcus aureus* and *Escherichia coli* were detected after treatment with FM (Figure 5). In addition, the morphological changes in *Staphylococcus aureus* and *Escherichia coli* were observed by SEM. As expected, FM treatment resulted in cell deformation and collapse (Figure 6). All this evidence illustrated that the anti-microbial peptides and organic acids in FM interacted with bacterial membranes and exerted anti-bacterial activity by inducing membrane depolarization and increasing membrane permeability.

## 4. Materials and Methods

### 4.1. Materials

Skim milk was purchased from Saputo Lotion Co., Ltd. (Shanghai, China). *Lactobacillus plantarum* was deposited in the China Center for Type Culture Collection (CCTCC No: M20232264). *Staphylococcus aureus* (ATCC 6538) and *Escherichia coli* (ATCC 8099) were received from Guangdong Huankai Microbial Technology Co., Ltd. (Guangzhou, China). Protease was bought from Xiasheng (Beijing) Biotechnology Development Co., Ltd. (Beijing, China). The medium de Man–Rogosa–Sharpe (MRS) broth was obtained from Qingdao Haibo Biotechnology Co., Ltd. (Qingdao, China). Glucose, yeast powder, sodium chloride, peptone, potassium chloride (KCl), phenol, sulfuric acid, trichloroacetic acid (TCA), and ethylenediamine tetraacetic acid (EDTA) were acquired from Sinopharm Chemical Reagent Co., Ltd. (Shanghai, China). 4-hydroxyethyl piperazine ethanesulfonic acid (HEPES), phosphate-buffered saline (PBS), and the BCA Protein Assay Kit were obtained by Biyuntian Biotechnology (Shanghai, China). The dyes of 3,3′-Dipropylthiadicarbocyanine Iodide (DiSC3 (5)), SYTO 9 Green Fluorescent Nucleic Acid Stai (SYTO 9), and Propidium Iodide (PI) were purchased from Shanghai Haoyuan Biomedical Technology Co., Ltd. (Shanghai, China).

### 4.2. Preparation of PM and FM

#### 4.2.1. Preparation of PM

A 10 kDa membrane separation device (SA-UF-QS1812, Sepp Biotechnology Engineering, Shanghai, China) was used to remove lactose from milk. Afterwards, 0.5% protease was added into the milk dispersion, and the pH was adjusted to 8.0, followed by hydrolyzing at 50 °C. After 3.5 h, the protease was inactivated at 90 °C for 10 min. After cooling down to room temperature, the pH of the dispersion was adjusted to 5.5 to prepare the enzymatic hydrolysate of skim milk. The supernatant of enzymatic hydrolysate was centrifugated at 10,000 rpm for 10 min and then filtrated through a 0.22 μm membrane. After freeze-drying, PM was obtained and kept at −20 °C.

#### 4.2.2. Preparation of FM

The *Lactobacillus plantarum*, stored at −80 °C, was rapidly thawed at 37 °C. Following that, it was inoculated in MRS broth at 2.0% inoculum and subcultured twice before conducting fermentation.

The PM was placed in a 5 L fermentation reactor (T&J-Intelli-Ferm A, T&J Bio-engineering, Shanghai, China), in which the final concentrations of 2% glucose and 0.5% yeast powder were added. After autoclaving, the seed medium was transferred to the fermentation reactor with the final concentration being 5%. The suspension was maintained at 37 °C with constant stirring (100 rpm) and a pH of 5.5 ± 0.2 (adjusted with 5 M NaOH) for 12 h. Then, the fermentation broth was subjected to the same post-treatment as the PM to prepare FM.

### 4.3. Measurement of Protein, Peptide, and Amino Acid Contents

The protein content was determined using the BCA Protein Assay Kit directly, while the peptide and amino acid contents were tested by the BCA Protein and Amino Acid Assay Kit, respectively. Before testing, the samples were pre-treated in the following manner: PM/FM was added to 5% TCA and set aside for 20 min, and then centrifuged at 3500 rpm for 10 min to obtain the supernatant, which was followed by filtering with a 0.22 μm membrane.

### 4.4. Measurement of Organic Acid Content

High-Performance Liquid Chromatography (HPLC, Arc, Waters Corporation, Milford, MA, USA) was employed to analyze the proportions of organic acid in PM and FM using a chromatographic column (COSMOSIL 5C18-AR-II column, Greenherbs Science and Technology, Beijing, China). The mobile phases consisted of methanol (A) and 0.1% phosphoric acid (B), with a flow rate of 0.6 mL/min. The gradient settings were 100% A at 0–5 min and 90% A at 5–15 min. The detection wavelength was 210 nm. Peak identification was confirmed by the retention time and the spectra of standard substances, including lactic acid and acetic acid.

### 4.5. Measurement of Polysaccharide Content

The phenol–sulfuric acid method according to the description of Wu et al. was adopted to assess the polysaccharide content [26]. An amount of 1 mL of the sample was mixed with 1 mL of 5% phenol solution and 5 mL of concentrated sulfuric acid. Afterwards, the mixture was incubated in a boiling water bath for 15 min, and the absorbance value of the solution at the wavelength of 490 nm (OD_490_) was measured. Glucose, utilized to establish the standard curve, was diluted to different concentrations, ranging from 0.02 to 0.08 mg/mL. The standard equation based on the OD_490_ values was y = 11.85 x + 0.0049, R^2^ = 0.9907.

### 4.6. Molecular Weight Distribution of Peptides

HPLC (Waters 2695, Waters Corporation, Milford, MA, USA) equipped with a size-exclusion column (TSKgel 2000 SWXL, Tosoh, Tokyo, Japan) was applied to determine the molecular weight distribution of PM and FM. Chromatographic analysis was conducted through a mobile phase composed of three components, acetonitrile (A), water (B), and trifluoroacetic acid (C), with a volume ratio of 40:60:0.1 and a flow rate of 0.5 mL/min. Additionally, the detection wavelength was 220 nm. A series of standards with known molecular weights, including cytochrome C (12,384 Da), aprotinin (6500 Da), bacitracin (1422 Da), Gly-gly-tyr-arg (451 Da), and Gly-gly-gly (189 Da), were exploited to identify the molecular weight of the separated compounds.

### 4.7. Peptidomics Analysis of PM and FM

After the sample was desalted with a self-filling column, the peptides were separated by a nanocolumn (Acclaim PepMap C18, Dr. Maisch GmbH, Tübingen, Baden-Württemberg, Germany) at a rate of 600 nL/min. The mobile phase comprised 0.1% formic acid (A) and 0.1% formic acid plus 80% acetonitrile (B), with a gradient of 4% to 8% B for 0–3 min, 8% to 28% B for 3–89 min, 28% to 40% B for 89–109 min, 40% to 95% B for 109–110 min, and 95% to 95% B for 110–120 min. Afterwards, peptides were examined by MS (Q Exactive, Thermo Fisher Scientific, Waltham, MA, USA), and the spray voltage and capillary temperature were 2.2 kV and 320 °C, respectively.

Peaks Studio10.6. was used to analyze the obtained data. The proportion of each peptide segment in FM was determined by dividing the peak area of each peptide segment with the total peak area. The activity prediction and score of the peptides in FM were realized via PeptideRanker (http://distilldeep.ucd.ie/PeptideRanker/, accessed on 28 November 2023), and the possible biological activity of peptides with the predicted activity scores > 0.5 was identified through BIOPEP (https://biochemia.uwm.edu.pl/biopep-uwm/, accessed on 28 November 2023) [27,28].

### 4.8. Anti-Oxidant Activity

The DPPH radical scavenging rate described by Adolfsson et al. was used to assess anti-oxidant activity with a slight modification [29]. Amounts of 10 μL of the samples with different concentrations (5, 25, 50, 100, 150, and 200 mg/mL), 90 μL of deionized water, and 100 μL of 0.8 mg/mL DPPH in ethanol were gently mixed at room temperature for 30 min. The absorbance of the mixture at 517 nm (OD_517_) was measured by a microplate reader (SpectraMax M2, Molecular Devices, Sunnyvale, CA, USA).

The formula for calculating the DPPH radical scavenging rate is as follows:

(1) $$\mathrm{DPPH}\%=\left(1-\frac{{\mathrm{OD}}_{A}-{\mathrm{OD}}_{\mathrm{AB}}}{{\mathrm{OD}}_{B}-{\mathrm{OD}}_{\mathrm{BB}}}\right)\times 100\%$$

OD_A_ and OD_B_ are the OD_517_ values of the test samples and the control without samples, respectively, and OD_AB_ and OD_BB_ are the OD_517_ values of the samples and the control without DPPH.

### 4.9. Anti-Bacterial Activity

#### 4.9.1. The MIC Values of FM

The MIC was determined through a modified method of Zhu et al. [30]. Briefly, LB medium was prepared by mixing 10 g/L of peptone, 5 g/L of yeast extract, and 10 g/L of sodium chloride solution, with the pH at 7.0–7.2. *Staphylococcus aureus* and *Escherichia coli* cultured in LB broth for 12 h were collected by centrifugation and diluted with LB broth to 10^6^ CFU/mL. The bacterial suspensions and samples were added to a sterile 96-well plate in equal proportions. The 96-well plate was placed in a bacterial incubator at 37 °C for 24 h. Afterwards, the absorbance of each well at a wavelength of 600 nm (OD_600_) was tested at 0 h and after incubation for 24 h. The MIC corresponded to the concentration where the OD_600_ difference between 24 h and 0 h was less than 0.05.

#### 4.9.2. Bacterial Survival Analysis

After being cultured for 12 h, *Staphylococcus aureus* and *Escherichia coli* were collected and diluted to 10^6^ CFU/mL. The sample solution or the corresponding concentration of organic acids was then added into equal amounts of bacterial dispersion. After incubation at 37 °C for 24 h, the OD_600_ value was measured.

The percentage of bacterial survival rate was calculated according to the following formula:

(2) $$\mathrm{Bacterial}\;\mathrm{Survival}\%=\frac{{\mathrm{OD}}_{S}-{\mathrm{OD}}_{\mathrm{SB}}}{{\mathrm{OD}}_{C}-{\mathrm{OD}}_{\mathrm{CB}}}\times 100\%$$

OD_S_ and OD_C_ are the OD_600_ values of the experimental group and control group, respectively. OD_SB_ and OD_CB_ represent the OD_600_ value of 100 μL of LB broth plus 100 μL of the corresponding sample solution or plus 100 μL of LB broth.

#### 4.9.3. Cytoplasmic Membrane Depolarization

According to the method described by Wang et al., the effects of PM and FM on cell membrane depolarization were evaluated using the dye DiSC3 (5) [31]. After culturing to the logarithmic growth phase, *Staphylococcus aureus* and *Escherichia coli* were washed twice and suspended in a mixture of HEPES buffer (5 mM), glucose (20 mM), and KCl (100 mM). For *Escherichia coli*, the permeabilization of the outer film and dye absorption was achieved by adding 0.2 mM EDTA [32]. Amounts of 50 μL of bacterial suspension with a colony count of 10^7^ CFU/mL plus 50 μL of 4 μM DiSC3 (5) were added to the 96-well plate and incubated under dark conditions for 10 min to allow maximum dye absorption. Following this, 100 μL of 400 mg/mL of PM/FM was added into duplicate wells. The plate was then put into the microplate reader rapidly to monitor the fluorescence intensity at the excitation wavelength of 622 nm and the emission wavelength of 670 nm, recording data every minute for 30 min. HEPES buffer and 25% Triton X-100 were used as negative and positive controls, respectively. The fluorescence intensity of 50 µL of the buffer, 50 µL of DiSC3 (5), and 100 µL of the sample was used as the blank control to determine whether the sample interfered with the fluorescence measurement.

#### 4.9.4. Bacterial Cell Membrane Permeability

Bacterial cell membrane integrity was tested using the dye SYTO 9 and PI [33]. The bacteria (10^7^ CFU/mL) were resuspended in PBS and then incubated with the samples at 37 °C for 12 h. After washing with 0.85% saline twice, the bacteria were incubated with a mixture of 5 μM SYTO 9 and 30 μM PI for 15 min. Laser scanning confocal microscopy (TCS SP8, Leica, Wetzlar, Hesse, Germany) was utilized to observe the fluorescence images at an excitation wavelength of 488 nm. ImageJ software 1.54g was applied to semi-quantitatively analyze the total fluorescence intensity of PI and SYTO 9.

#### 4.9.5. Observation of Bacteria Morphology by SEM

Bacteria (10^7^ CFU/mL) were first co-cultured with 200 mg/mL samples for 3 h and 24 h at 37 °C. Afterwards, 50 μL of culture solution was dropped onto the clean silicon wafer and left to air-dry. Subsequently, the silicon wafer was immersed in a solution containing 2.5% glutaraldehyde for 2 h, washed with PBS, and then dehydrated in a gradient of 30%, 50%, 70%, and 100% ethanol for 15 min, respectively. Finally, the gold-plated silicon wafers were subjected to SEM analysis [34].

### 4.10. Statistical Analysis

All the data are presented in the form of averages of three independent trials ± standard deviations (SD). ANOVA and Duncan’s test in IBM SPSS Statistics 27 were applied to analyze the differences between groups where *p* < 0.05 was regarded as being statistically significant. Meanwhile, graphs were produced using Origin 2021b software.

## 5. Conclusions

In this study, the peptide composition of FM was significantly altered compared to that of PM. The fermentation process produced a variety of new oligopeptides, which could be readily absorbed and metabolized by humans. In addition, FM exhibited a higher anti-oxidant capacity than PM. More importantly, FM displayed outstanding anti-bacterial activity against both Gram-positive and Gram-negative bacteria, whereas PM did not show such efficacy. The anti-bacterial properties of FM were primarily attributed to the newly produced peptides and, to a lesser extent, to the organic acids produced during fermentation. Additionally, the potential anti-bacterial mechanisms of FM were investigated, which may involve the cytomembrane depolarization and the increased cytomembrane permeability. In summary, due to the bioactivity and safety of FM, it has the potential to serve as an anti-oxidant and anti-microbial agent in the food industry, as well as in the pharmaceutical and cosmetic industries to address diseases or skin issues caused by oxidative stress or bacterial infections.

## Figures and Tables

**Table 1:** The total content of protein, peptide, amino acid, organic acid, and polysaccharide in PM and FM.

Sample	Protein (%)	Peptide (%)	Amino Acid (%)	Organic Acid (%)	Polysaccharide (%)
PM	3.84 ± 1.04	39.94 ± 1.74	1.93 ± 0.06	N.D.	12.24 ± 0.09
FM	1.36 ± 0.44	30.76 ± 1.95	2.12 ± 0.15	33.73 ± 2.83	11.98 ± 0.09

N.D. indicates that the component was not detected.

**Table 2:** Identification of the major potential anti-oxidative and anti-bacterial peptides derived from FM.

NO.	Protein Name	Peptide Sequences	Molecular Weight (Da)	Predicted Activity Score	Bioactive	Proportion (%)
1	β-casein	SLVYPFPGPIH	1225.65	0.76	Anti-oxidative	0.34
2	β-casein	LLYQEPVLGPVRGPFPIIV	2106.22	0.72	Anti-oxidative	0.04
3	α-S2-casein	INPSKENLCSTFCKEVVRN	2294.11	0.69	Anti-oxidative	2.97
4	α-S2-casein	INPSKENLCSTFCKEVVR	2180.07	0.65	Anti-oxidative	0.81
5	β-casein	KEMPFPKYPVEPF	1607.81	0.64	Anti-oxidative; Anti-bacterial	2.75
6	α-S2-casein	INPSKENLCSTFCKE	1825.83	0.64	Anti-oxidative	1.40
7	α-lactalbumin	HKALCSEKLDQWLCEKL	2157.07	0.64	Anti-bacterial	0.21
8	α-S2-casein	PSKENLCSTFCKEVVRNA	2138.03	0.61	Anti-oxidative	0.07
9	β-casein	VYPFPGPIHNSLPQ	1564.80	0.61	Anti-oxidative	0.03
10	α-S2-casein	SKENLCSTFCKEVVRN	1969.93	0.59	Anti-oxidative	1.87
11	α-S2-casein	SKENLCSTFCKEVVR	1855.89	0.55	Anti-oxidative	0.19
12	β-casein	PKHKEMPFPKYPVEPF	1970.01	0.54	Anti-oxidative; Anti-bacterial	1.60
13	β-casein	PKHKEMPFPKYP	1497.78	0.53	Anti-bacterial	5.94
14	β-casein	LPVPQKAVPYPQRDMP	1834.98	0.53	Anti-oxidative	0.45
15	β-casein	KHKEMPFPKYPVEPF	1872.96	0.51	Anti-oxidative; Anti-bacterial	41.99
16	α-S2-casein	SKENLCSTFCKEVVRNA	2040.97	0.51	Anti-oxidative	0.29

The proportion is defined as the ratio of the peak area of each peptide to the total peak area of all peptides detected in FM.

**Table 3:** The MIC values of FM.

Type	Strains	MIC (mg/mL)
Gram-positive bacteria	*Staphylococcus aureus*	200
Gram-negative bacteria	*Escherichia coli*	150

**Table 4:** Fluorescence intensity of samples with the dye DiSC3 (5).

Samples	PM	FM	Control	25% TritonX-100
Fluorescence intensity	1039.28 ± 99.08	395.38 ± 24.09	166.61 ± 7.80	585.37 ± 34.86

## Data Availability

The data presented in this study are available upon request from the corresponding authors.

## Supplementary Materials

The following supporting information can be downloaded at https://www.mdpi.com/article/10.3390/molecules29163800/s1, Table S1: Prediction activity scores of peptides in FM.

## References

[1] Lin T., Meletharayil G., Kapoor R., Abbaspourrad A. (2021). Bioactives in Bovine Milk: Chemistry, Technology, and Applications. Nutr. Rev..

[2] Cao R., Li W., Zhang J., Bao X., Feng H., Sun J., Liu X., Sun L. (2024). Milk Casein Hydrolysate Peptides Regulate Starch Digestion through Inhibition of α-glucosidase: An Insight into the Active Oligopeptide Screening, Enzyme Inhibition Behaviors, and Oligopeptide-enzyme Binding Interactions. Food Hydrocoll..

[3] Wakabayashi H., Yamauchi K., Takase M. (2006). Lactoferrin Research, Technology and Applications. Int. Dairy J..

[4] Kazimierska K., Kalinowska-Lis U. (2021). Milk Proteins—Their Biological Activities and Use in Cosmetics and Dermatology. Molecules.

[5] Esmaeilpour M., Ehsani M.R., Aminlari M., Shekarforoush S., Hoseini E. (2016). Antimicrobial Activity of Peptides Derived from Enzymatic Hydrolysis of Goat Milk Caseins. Comp. Clin. Pathol..

[6] Kang L., Han T., Cong H., Yu B., Shen Y. (2022). Recent Research Progress of Biologically Active Peptides. Biofactors.

[7] Liu S., Zhao L., Li M., Zhu Y., Liang D., Ma Y., Sun L., Zhao G., Tu Q. (2024). Probiotic Bacillus as Fermentation Agents: Status, Potential Insights, and Future Perspectives. Food Chem. X.

[8] Yang H.Y., Han L., Lin Y.Q., Li T., Wei Y., Zhao L.H., Tong X.L. (2023). Probiotic Fermentation of Herbal Medicine: Progress, Challenges, and Opportunities. Am. J. Chin. Med..

[9] Begunova A.V., Savinova O.S., Glazunova O.A., Moiseenko K.V., Rozhkova I.V., Fedorova T.V. (2020). Development of Antioxidant and Antihypertensive Properties During Growth of Lactobacillus helveticus, Lactobacillus rhamnosus and Lactobacillus reuteri on Cow’s Milk: Fermentation and Peptidomics Study. Foods.

[10] Sun L., Gong M., Lv X., Huang Z., Gu Y., Li J., Du G., Liu L. (2020). Current Advance in Biological Production of Short-chain Organic Acid. Appl. Microbiol. Biotechnol..

[11] Garnier L., Penland M., Thierry A., Maillard M.B., Jardin J., Coton M., Leyva Salas M., Coton E., Valence F., Mounier J. (2020). Antifungal Activity of Fermented Dairy Ingredients: Identification of Antifungal Compounds. Int. J. Food Microbiol..

[12] Izawa N., Sone T., Anazawa H., Shimizu S. (2014). Cosmetic Ingredients Fermented by Lactic Acid Bacteria. Microbial Production.

[13] Picon A., García-Casado M.A., Nuñez M. (2010). Proteolytic Activities, Peptide Utilization and Oligopeptide Transport Systems of Wild Lactococcus lactis Strains. Int. Dairy J..

[14] Aguilar-Toalá J.E., Santiago-López L., Peres C.M., Peres C., Garcia H.S., Vallejo-Cordoba B., González-Córdova A.F., Hernández-Mendoza A. (2017). Assessment of Multifunctional Activity of Bioactive Peptides Derived from Fermented Milk by Specific Lactobacillus plantarum Strains. J. Dairy Sci..

[15] Wang Z., Tang H., Liu G., Gong H., Li Y., Chen Y., Yang Y. (2023). Compound Probiotics Producing Cellulase Could Replace Cellulase Preparations During Solid-state Fermentation of Millet Bran. Bioresour. Technol..

[16] Nutten S., Schuh S., Dutter T., Heine R.G., Kuslys M. (2020). Design, Quality, Safety and Efficacy of Extensively Hydrolyzed Formula for Management of Cow’s milk Protein Allergy: What are the Challenges?. Adv. Food Nutr. Res..

[17] Gooran N., Tan S.W., Frey S.L., Jackman J.A. (2024). Unraveling the Biophysical Mechanisms of How Antiviral Detergents Disrupt Supported Lipid Membranes: Toward Replacing Triton X-100. Langmuir.

[18] Ribeiro M.M.B., Franquelim H.G., Castanho M.A.R.B., Veiga A.S. (2007). Molecular Interaction Studies of Peptides Using Steady-state Fluorescence Intensity. Static (De)quenching Revisited. J. Pept. Sci..

[19] Cui Q., Duan Y., Zhang M., Liang S., Sun Y., Cheng J., Guo M. (2022). Peptide Profiles and Antioxidant Capacity of Extensive Hydrolysates of Milk Protein Concentrate. J. Dairy Sci..

[20] Stobiecka M., Król J., Brodziak A. (2022). Antioxidant Activity of Milk and Dairy Products. Animals.

[21] Cirrincione S., Luganini A., Lamberti C., Manfredi M., Cavallarin L., Giuffrida M.G., Pessione E. (2021). Donkey Milk Fermentation by Lactococcus lactis subsp. cremoris and Lactobacillus rhamnosus Affects the Antiviral and Antibacterial Milk Properties. Molecules.

[22] Pihurov M., Păcularu-Burada B., Cotârleț M., Bahrim G.E. (2022). Tailoring the Optimized Fermentation Conditions of SCOBY-Based Membranes and Milk Kefir Grains to Promote Various Functional Properties. Foods.

[23] Singh B.P., Bhushan B., Vij S. (2020). Antioxidative, ACE Inhibitory and Antibacterial Activities of Soy Milk Fermented by Indigenous Strains of Lactobacilli. Legume Sci..

[24] Wei C., Cui P., Liu X. (2023). Antibacterial Activity and Mechanism of Madecassic Acid Against Staphylococcus aureus. Molecules.

[25] Fan Q., Yan C., Shi C., Xu Y., Ma Y., Zhang C., Peng X., Xia X. (2019). Inhibitory Effect of Coenzyme Q0 on the Growth of Staphylococcus aureus. Foodborne Pathog. Dis..

[26] Wu L., Gao Y., Ren W.C., Su Y., Li J., Du Y.Q., Wang Q.H., Kuang H.X. (2022). Rapid Determination and Origin Identification of Total Polysaccharides Contents in Schisandra Chinensis by Near-infrared Spectroscopy. Spectrochim. Acta Part A Mol. Biomol. Spectrosc..

[27] Minkiewicz P., Iwaniak A., Darewicz M. (2019). BIOPEP-UWM Database of Bioactive Peptides: Current Opportunities. Int. J. Mol. Sci..

[28] Kurgan L., Mooney C., Haslam N.J., Pollastri G., Shields D.C. (2012). Towards the Improved Discovery and Design of Functional Peptides: Common Features of Diverse Classes Permit Generalized Prediction of Bioactivity. PLoS ONE.

[29] Adolfsson K.H., Huang P., Golda-Cepa M., Xu H., Kotarba A., Hakkarainen M. (2023). Scavenging of DPPH by Persistent Free Radicals in Carbonized Particles. Adv. Sustain. Syst..

[30] Chen H., Zhong Q. (2018). Antibacterial Activity of Acidified Sodium Benzoate against Escherichia coli O157: H7, Salmonella enterica, and Listeria Monocytogenes in Tryptic Soy Broth and on Cherry Tomatoes. Int. J. Food Microbiol..

[31] Wang X., Feng L., Li M., Dong W., Luo X., Shang D. (2023). Membrane-active and DNA Binding Related Double-action Antimycobacterial Mechanism of Antimicrobial Peptide W3R6 and Its Synthetic Analogs. Biochim. Biophys. Acta (BBA) Gen. Subj..

[32] Zhao X., Luo W., Wang L., Zhu C., Xue X., Xia X., Wu X., Bai Y., Hu J. (2023). Antimicrobial Peptide Mastoparan X Has Good Activity Against Escherichia coli in Vitro and Alleviates Its Pathogenicity in Mice. Transl. Med. Commun..

[33] Boix-Lemonche G., Lekka M., Skerlavaj B. (2020). A Rapid Fluorescence-Based Microplate Assay to Investigate the Interaction of Membrane Active Antimicrobial Peptides with Whole Gram-Positive Bacteria. Antibiotics.

[34] Senthil B., Devasena T., Prakash B., Rajasekar A. (2017). Non-cytotoxic Effect of Green Synthesized Silver Nanoparticles and Its Antibacterial Activity. J. Photochem. Photobiol. B Biol..

