# Supplementary material for: Improved Anti-Oxidant and Anti-Bacterial Capacities of Skim Milk Fermented by Lactobacillus plantarum
Source: Molecules. 2024 Aug 10;29(16):3800. doi: 10.3390/molecules29163800 (PMC11356925; doi:10.3390/molecules29163800)
Supplement: Supplementary file 1 [file molecules-29-03800-s001.zip › molecules-3104643-supplementary.pdf]

Improved Anti-oxidant and Anti-bacterial Capacities of Skim Milk Fermented by *Lactobacillus plantarum*

Ying Wang[1], BingTian Zhao[1], Yun Ding[2], Nan Liu[2,*], Cheng Yang[1], Yajuan Sun[1,*]

[1] Key Laboratory of Synthetic and Biological Colloids, Ministry of Education, School of Chemical and Material Engineering, Jiangnan University, Wuxi 214122, China; wy2133590196@163.com (Y.W.); btzhao@jiangnan.edu.cn (B.Z.); cyang@jiangnan.edu.cn (C.Y.)
[2] Hangzhou Island Xingqing Biotechnology Co., Ltd., Hangzhou 310023, China; dingyun@mompick.com
* Correspondence: liunan@mompick.com (N.L.); cmsun@jiangnan.edu.cn (Y.S.)

Table S1. Prediction activity scores of peptides in FM.

No.	Predicted Activity Score	Peptide Sequences
1	0.890369	PFPGPIPN
2	0.82043	FVAPFPEVF
3	0.760659	PVVVPPFL
4	0.760295	SLVYPFPGPIH
5	0.751054	GGVSLPEWVCTTFHT
6	0.731187	SFSDIPNPIG
7	0.716627	LLYQEPVLGPVRGPFPIIV
8	0.685692	INPSKENLCSTFCKEVVRN
9	0.654729	INPSKENLCSTFCKEVVR
10	0.643289	KEMPFPKYPVEPF
11	0.638183	INPSKENLCSTFCKE
12	0.636286	HKALCSEKLDQWLCEKL
13	0.622409	VVVPPFL
14	0.609199	PSKENLCSTFCKEVVRNA
15	0.607549	VYPFPGPIHNSLPQ
16	0.600786	SLPEWVCT
17	0.585556	SKENLCSTFCKEVVRN
18	0.568686	GGVSLPEWVCTTFHTS
19	0.546424	SKENLCSTFCKEVVR
20	0.538298	PKHKEMPFPKYPVEPF
21	0.534367	PKHKEMPFPKYP
22	0.529251	LPVPQKAVPYPQRDMP
23	0.522457	APSFSDIPNPIGS
24	0.512202	KHKEMPFPKYPVEPF
25	0.51211	SKENLCSTFCKEVVRNA

26	0.488408	KHKEMPFPKYP
27	0.461541	DAPSFSDIPNPIGS
28	0.428512	NLCSTFCKEVVRN
29	0.425836	SLPQNIPPLTQTP
30	0.421748	SLPEWVCTTFHTS
31	0.418947	FLQPEVM
32	0.41858	QTQSLVYPFP
33	0.400357	AVPITPTL
34	0.393131	NICNISCDKFLDDDLTDDIM
35	0.379916	NICNISCDKFLDD
36	0.377085	ISLLDAQSAPLRVY
37	0.370899	SFSDIPNPIGSEN
38	0.366839	ELQDKIHPF
39	0.32516	LKPTPEGDLEILLQ
40	0.320997	GTQYTDAPSFSDIPNPIGSEN
41	0.307896	KHKEMPFPKYPVEP
42	0.304101	AINPSKENLCSTFCKEVVRNA
43	0.302831	YTRVVWCAVGPEEQKKCQQW
44	0.260468	AASDISL
45	0.254489	GTQYTDAPSFSDIPNPIG
46	0.22331	TQYTDAPSFSDIPNPIG
47	0.200607	QYTDAPSFSDIPNPIGSEN
48	0.200281	QEQNQEQPIRCE
49	0.1142	KFQSEEQQQTEDELQDKIHPF
50	0.105977	SKVLPVPQKAVPYPQRDMPIQAF
51	0.0806835	EGDYVLFHHEGGVDVGDVDAK
52	0.074425	VHTVEDYQAIVDAEWNILYDK
53	0.024434	ITAFVPNDGCLNFIEENDEVLVAGFGR
54	0.024434	ITAFVPNDGCLNFIEENDEVLVAGFGR
